# Neutrophil lymphocyte ratio (NLR): A well assessment tool of glycemic control in type 2 diabetic patients

**DOI:** 10.12669/pjms.336.12900

**Published:** 2017

**Authors:** Mazhar Hussain, Muhammad Zafar Majeed Babar, Lubna Akhtar, Muhammad Shahbaz Hussain

**Affiliations:** 1 Dr. Mazhar Hussain, MBBS, M.Phil. Department of Pharmacology, Sheikh Zayed Medical College & Hospital, Rahim Yar Khan, Pakistan; 2 Dr. Muhammad Zafar Majeed Babar, MBBS, FCPS. Department of Medicine, Sheikh Zayed Medical College & Hospital, Rahim Yar Khan, Pakistan; 3 Dr. Lubna Akhtar, MBBS, FCPS. Department of Pharmacology, Sheikh Zayed Medical College & Hospital, Rahim Yar Khan, Pakistan; 4 Dr. Muhammad Shahbaz Hussain, MBBS, M.Phil. Department of Microbiology, Sheikh Zayed Medical College & Hospital, Rahim Yar Khan, Pakistan

**Keywords:** NLR, Type 2 diabetes, CRP, HbA1c

## Abstract

**Background and Objective:**

Increased neutrophil lymphocyte ratio (NLR) is a marker as well as predictor of various cardiac and non cardiac disorders. Our aim was to assess the relationship between NLR and different level of glycemic control in type 2 diabetic patients.

**Methods:**

An observational study was conducted at diabetic clinic of Sheikh Zayed Medical College/Hospital, Rahim Yar Khan from September 2016 to February 2017 in which 330 type 2 diabetic patients were randomly divided in to three groups based upon diabetes control according to ADA criteria. Patients in group A with HbA1c ≤ 7% (excellent control), group B HbA1c 7.0-9.0 % (poor control) and group C HbA1c ≥ 9 %(worst control). Patients were assessed in terms of complete blood count and C - reactive protein.

**Results:**

As compared to excellent control (Group A) patients with worst control (Group C)showed a high leukocyte count (p.001), high neutrophil count (P.003) and lower lymphocyte count (P 0.44) while patients in poor control (Group B)did not differ significantly. Similarly value of NLR was also significantly higher in worst control (Group C) as compared to poor control(Group B) and excellent control (Group A) diabetes (4.3±2.8, 2.7±1.0 and2.0±0.5(p.001). NLR were found independent predictor of worst diabetes control (OR: 1.809, 95% CI: 1.459-2.401) along with fasting blood sugar (OR: 0.938, 95% CI: 0.995-0.982) and CRP (OR: 1.020, 95% CI: 1.003-1.028).

**Conclusion:**

Increased NLR level is associated with elevated HbA1c and poor glycemic control in patients of type 2 diabetes mellitus. It can be used as a disease monitoring tool during the follow up of diabetic patients.

## INTRODUCTION

Diabetes mellitus a chronic preventable metabolic disorder and is considered to be one of the silent killer all across the globe because most of the patient remain asymptomatic for years unless they develop complications. The burden of diabetes is on the rise in middle and lower income countries due to strong influence of urbanization, sedentary life style, nutritional and epidemiological transition.^1^ It is estimated that the rise of type 2 diabetes in South Asian countries is more than 150% between 2000 and 2035. Therefore drastic steps are required through various health awareness programs in order to control the escalating trends of diabetes in these countries to reduce disease burden.^2^

The increased number of circulating leukocytes not only reflects a presence of acute infection in the body but they have also a dominant inflammatory role in the pathogenesis of different phases of atherosclerosis from its start to advancement and ultimately complications in the form of eventual rupture of atherosclerosis plaque that leads to various cardiovascular disorders. Studies have shown that increased leukocyte counts are reliable markers of systemic inflammation and have diagnostic as well as prognostic value in patients of angina, myocardial infarction, stroke, peripheral vascular disease and micro and macro vascular complication associated with diabetes.^3^,^4^

Now a day’s there is more concern about neutrophil lymphocyte ratio NLR, a potential marker to determine inflammation in various cardiac and non-cardiac disorders because it has a superior predictive, diagnostic and discriminative ability than total WBC count. Moreover predictive value of NLR is comparable to various other inflammatory markers such as C – reactive protein (CRP), tumor necrosis factor (TNF-α) and interleukin (IL-6) in the detection of subclinical inflammation and endothelial dysfunction in various clinical studies.^5^,^6^ NLR, a traditional marker has various advantages over other inflammatory markers because of its wide availability, low cost, reliability, easy lab detection. Moreover NLR can be used as population screening, disease and drug monitoring tool on large scale basis. Even patients with increase NLR but normal TLC count could have increased risk of atherosclerosis related diseases.^7^-^9^

HbA1c is a widely available test which is usually advised by most clinicians to measures the long term glycemic control in diabetic patients over the period of previous 2-3 months.HbA1c also tells about the severity of hyperglycemia and considered to be a biomarker of risk factors for diabetes related micro and macro-vascular complications.^10^ However HbA1c usually do not predict ongoing inflammation and diabetes associated complications which are more precisely done by NLR. The present study was designed to assess relationship between NLR and glycemic control which was done by comparing NLR with HbA1c in terms of excellent, poor and worst diabetes control and compare them with CRP which is the most studied inflammatory markers.

## METHODS

This observational study was carried out at diabetic clinic of Sheikh Zayed Medical College/Hospital, Rahim Yar Khan from November 2016 to February 2017. All patients gave a written informed consent and study was approved by ethical committee. Initially 550 type 2 diabetic patients who came in diabetic clinic for regular follow up were recruited. On the basis of inclusion criteria, a total of 330 patients of both sexes were enrolled in the study. They were randomly divided in to three groups each contained 110 patients based on the severity of diabetes control according to ADA criteria. Patients in group A with HbA1c ≤ 7% termed as (excellent control), group B HbA1c 7-9% (poor control) and patients group C HbA1c ≥ 9% (worst control) respectively. A detailed history and general physical examination were undertaken to rule out any causes which affects inflammation such as vitals and any localizing signs. The exclusion criteria were any acute or chronic infections and inflammatory conditions for last two weeks. Patients with chronic heart failure, bleeding disorders, hematological disorders, dyslipidemia, hypertension, autoimmune diseases, cancers, severe liver and kidney disorders were excluded from the study. In addition detailed history was taken about smoking, alcohol use and drugs which effect inflammation like antidyslipidemic agents, non steroidal anti inflammatory drugs (NSAIDS), opiods analog, hormonal contraceptives, steroids, immuno-suppressive and immune- modulators drugs.

Blood sample was collected from the median cubital vein after an overnight fasting of 12 hours. Total leukocyte counts (TLC) were measured by automated hematology analyzer. Neutrophil lymphocyte ratios (NLR) were estimated by dividing the absolute neutrophil ratio to absolute lymphocyte ratio. HbA1c level was measured by high performance liquid chromatography using automated ion exchange method while CRP level was measured by spectro photometry method.

### Data Analysis

Statistical package for social sciences (SPSS-16) were used for data analysis. Data were presented as mean ± standard deviation for continuous variables and percentages for categorical variables. The independent sample t test or Mann- Whitney U test was used for the continuous variable while chi-square for the categorical variables. ANOVA or Kruskal-Wallis test were used to compare the three groups. Multivariate logistic regression analysis was used with significant independent variables in association with worst diabetes control. All significant variables in univariate analysis were included in the logistic regression model shown as odds ratio (OR) and 95% confidence interval (CIs). A 2-tailed p ≤ was deemed to be statistically significant.

## RESULTS

Demographic characteristics and hematological parameters at baseline of excellent control (Group A), poor control (Group B) and worst control (Group C) are shown in Table-I. There were no significant statistical differences in terms of age, sex, family history and duration of diabetes in three groups. However serum glucose (P=0.004) and BMI (P=0.002) were significantly higher in worst control (Group C). Hemoglobin and platelets count were similar in all study groups. As compared to excellent control (Group A), Patients with worst control (Group C) showed a high leukocyte count (p=0.001), high neutrophil count (P= 0.003) and lower lymphocyte count(P= 0.44) while patients with poor control (Group B) did not differ. The value of NLR was also significantly higher in worst control(Group C) as compared to poor control (Group B)and excellent control(Group A)(4.3±2.8,2.7±1.0 and2.0±0.5 respectively p.001) Table-I.

**Table-I:** Baseline characteristics of three groups.^a^

*Parameters*	*Group A n=110 (HbAIc <7%) Excellent control*	*Group B n=110 (HbAIc 7-9%) Poor control*	*Group C n=110 (HbAIc >9%) Worst control*	*P Value^b^*
Age(years)	62.6±13.0	64.6±15.9	60.4±12.7	0.226
Male (n-%)	52(74)	48(68)	54(77)	0.375
BMI (kg/m^2^)	26.5±2.8	27.2±3.2	29.2±3.8	0.002
Diabetes>10years (n-%)	14(20)	17(24)	19(27)	0.502
Family history of CAD (n-%)	15(21)	12(17)	16(23)	0.404
Glucose(mg/dl)	106±24.2	124±45.8	152.8±65.4	0.004
Hemoglobin g/dl	12.8±1.84	12.7±2.23	13.1±1.5	0.392
Platelets count×_10^9^/L_	245±85	254±76	249±80	0.390
WBC count ×_10^9^/L_	7.4±2.1	8.6±3.6	9.8±2.8	0.001
Neutrophil count×_10^9^/L_	4.6±2.4	5.6±3.2	7.4±2.8	0.001
Lymphocyte count×_10^9^/L_	2.3±0.8	2.3±0.7	2.0±0.9	0.44
NLR	2.0±0.5	2.7±1.0	4.3±2.8	0.001
CRP	0.8±0.92	2.62±0.42	4.43±0.54	0.003

BMI, body mass index; NLR, neutrophil to lymphocyte ratio; CRP, C-reactive protein; SD, standard deviation; ANOVA, analysis of variance

a Values are mean± SD or n(%)

b ANOVA, Chi-square test or Kruskal-Wallis

A multivariate analysis showed that high level of NLR was independent predictor of worst diabetes control (Group C)(OR: 1.809, 95% CI: 1.459-2.401) along with fasting blood sugar (OR: 0.938, 95% CI: 0.995-0.982) and CRP (OR:1.020, 95% CI: 1.003-1.028). Table-II

**Table-II:** Multivariate logistic regression analysis with significant variable in association with worst diabetic control.

	*Odds Ratio (%95 confidence interval)*	*P value*
NLR	1.809(1.459-2.401)	< 0.001
Glucose mg/dl	0.938(0.995-0.982)	.021
BMI	1.905(0.453-9.644)	.589
CRP	1.020(1.003-1.028)	.001

CI, NLR, neutrophil to lymphocyte ratio; BMI, body mass index; C-reactive protein

## DISCUSSION

In this study we noticed a strong correlation between NLR and HbA1c. This is perhaps the first observational study to see the impact of NLR on different level of glycemic control on the basis of HbA1c values in type 2 diabetic patients and compare them with CRP. NLR is easily calculated from routine blood test by dividing the absolute neutrophil ratio to absolute lymphocyte ratio. It is more reliable than complete blood count in detecting subclinical inflammation in cardiac and non cardiac disorders. Studies have shown that NLR is not an inflammatory marker but it has strong diagnostic as well as prognostic potential for atherosclerosis related vascular diseases in which diabetes mellitus is one of them.^11^,^12^

A dominant role of NLR was seen in various studies conducted on diabetic patients. Shiny et al^13^ and Lou et al^14^ revealed that increased NLR has strong association with glucose intolerance and insulin resistance in type 2 diabetic patients. Regarding diabetes related micro vascular complications studies showed that NLR has a reliable predictive marker of early stage diabetic nephropathy^15^, retinopathy^16^ and diabetic foot ulcer.^17^ Moreover increased NLR is predictor of major cardiovascular events in both acute coronary syndrome and coronary artery disease patients and is also associated with increased carotid artery intima-media thickness in type 2 diabetic patients.^18^,^19^

In spite of extensive clinical studies of NLR in diabetic patients. Limited Data is available to determine the effect of NLR on glycemic control. Selfil et al^20^ revealed that increase NLR may be associated with elevated HbA1c in type 2 diabetic patients. Shiny et al^13^ compared the NLR value with normal glucose tolerance (NGT), impaired glucose tolerance (IGT) and type 2 diabetes mellitus (DM) and found a significant correlation with IGT and DM. Oh et al^21^ and Demirtas et al^22^ demonstrated association between glycemic control and hematological indices in type 2 diabetic patients and concluded that NLR can be used as a marker of diabetic regulations and complications during the follow up period of diabetic patients.

The present study showed that hyperglycemia is strongly related to increase NLR (i.e. decrease lymphocyte count and increase lymphocyte count). Even in our study a strong positive correlation was found between NLR and CRP in patients with worst diabetes control. Elevated CRP relation with white blood cells for glycemic control has been investigated in number of clinical studies.^23^-^24^ Our study showed that NLR not only assess different level of glycemic control but its high level may also predict that patient with worst control diabetes is likely to develop complications in future. So NLR is not an inflammatory marker only but also a direct cause of cardiovascular disease in diabetic patients like CRP.

### Limitation of study

The main limitation of our study was that we cannot correlate different antidiabetic agents used by patients with NLR as some antidiabetic drugs have also anti-inflammatory activity in addition to glycemic control.

## CONCLUSION

NLR values are assessment tool of different level of glycemic control in type 2 diabetic patients. Its high value is a predictor of worst diabetic control. So it can be used as a disease monitoring tool during the follow up of diabetic patients.

## RECOMMENDATION

NLR is a routinely performed test and is cost effective than HbA1c. It should be recommended in diabetic patients in routine clinical setting not for glycemic control but also for prediction of diabetes related complications in future so that effective measures should be taken in order to prevent these complications.

## References

[1] Hu FB (2011). Globalization of diabetes. Diabetes Care.

[2] Nanditha A, Ma RC, Ramachandran A, Snehalatha C, Chan JC, Chia KS (2016). Diabetes in Asia and the Pacific:implications for the global epidemic. Diabetes Care.

[3] Madjid M, Fatemi O (2013). Components of the complete blood count as risk predictor for coronary heart disease:a review. Tex Heart I J.

[4] Tong PC, Chan WB, Lee KF, Lo MK, So WY, Chan NN (2004). White blood cells count is associated with macro and microvascular inflammation complications in Chinese patients with type 2 diabetes. Diabetes Care.

[5] Ozturk C, Balta S, Balta I, Demirkol S, Celik T, Turker T (2015). Neutrophil lymphocyte ratio and carotid intima media thickness in patients with behcet disease without cardiovascular involvement. Angiology.

[6] Turkmen K, Guney I, Yerlikaya EH, Tonbul HZ (2012). The relationship between neutrophil to lymphocyte ratio and inflammation in end stage renal disease patients. Ren Fail.

[7] Balta S, Celik T, Mikhailidis DP, Ozturk C, Demirkol S, Aparci M (2016). The relationship between atherosclerosis and the neutrophil lymphocyte ratio. Clin Appl Thromb Hemost.

[8] Imtiaz F, Shafique K, Mirza SS, Ayoob Z, Vart P, Rao S (2012). Neutrophil lymphocyte ratio as a measure of systemic inflammation in prevalent chronic disease in Asian population. International Arch Med.

[9] Balta S, Kurtoglu E, Kucuk U, Demirkol S, Ozturk C (2014). Neutrophil lymphocyte ratio as an important assessment tool. Expert Rev Cardiovasc Ther.

[10] Lyons TJ, Basu A (2012). Biomarkers in diabetes:hemoglobin A1c, vascular and tissue markers. Translational Res.

[11] Horne BD, Anderson JL, John JM, Weaver A, Bair TL, Jensen KR (2005). Which white blood cell subtypes predict increased cardiovascular risk?. J Am Coll Cardiol.

[12] Bhat T, Teli S, Rijal J, Bhat H, Raza M, Khoueiry G (2013). Neutrophil to lymphocyte ratio and cardiovascular diseases:a review. Expert Rev Cardiovasc Ther.

[13] Shiny A, Bibin YS, Shanthirani CS, Regin BS, Anjana RM, Balasubramanyam M (2014). Association of neutrophil lymphocyte ratio with glucose intolerance:an indicator of systemic inflammation in patients with type 2 diabetes. Diabetes Technol Ther.

[14] Lou M, Luo P, Tang R, Peng Y, Yu S, Huang W (2015). Relationship between neutrophil lymphocyte ratio and insulin resistance in newly diagnosed type 2 diabetes mellitus patients. BMC Endocr Disord.

[15] Huang W, Haung J, Liu Q, Lin F, He Z, Zeng Z (2015). Neutrophil -lymphocyte ratio a reliable predictive marker of early stage diabetic nephropathy. Clin Endocrinol.

[16] Wang RT, Zhang JR, Li Y, Liu T, Yu KJ (2015). Neutrophil lymphocyte ratio is associated with arterial stiffness in diabetic retinopathy in type 2 diabetes. J Diabetes Complications.

[17] Kahraman C, Yumun G, Kahraman NK, Namdar ND, Cosgun S (2014). Neutrophil lymphocyte ratio in diabetes mellitus patients with and without diabetic foot ulcer. Eur J Med Sci.

[18] Azab B, Chainani V, Shah N, McGinn JT (2013). Neutrophil lymphocyte ratio as a predictor of major cardiovascular events among diabetic population:a 4 years follow up study. Angiology.

[19] Li X, Shen J, Lu Z, Chen M, Fang X, Wang G (2017). High neutrophil to lymphocyte ratio is associated with increased carotid artery intima-media thickness in type 2 diabetes. J Diabetes Invest.

[20] Selfil F, Ulutas KT, Dokuyucu R, Sumbul AT, Yengil E, Yagiz AE (2014). Investigation of neutrophil lymphocyte ratio and blood glucose regulation in patients with type 2 diabetes mellitus. J Int Med Res.

[21] Oh Y, Kwon GC, Koo SH, Kim J (2016). Association between glycemic control and hematolical indices in type 2 diabetic patients. Laboratory Medicine Online.

[22] Demirtas L, Degirmenci H, Akbas EM, Ozcicek A, Timuroglu A, Gurel A (2015). Association of hematological indices with diabetes, impaired glucose regulation and microvascular complications of diabetes. Int J Clin Exp Med.

[23] Xu Y, Zhao Z, Li X, Bi Y, Xu M, Ning M (2011). Relationship between c-reactive protein, white blood cell count and insulin resistance in Chinese populations. Endocrine.

[24] Oda E (2013). High sensitivity C-reactive protein and white blood cells counts equally predict development of metabolic syndrome in a Japanese health screening population. Acta Diabetologica.

